# Sorry, Not Sorry: Effects of Different Types of Apologies and Self-Monitoring on Non-verbal Behaviors

**DOI:** 10.3389/fpsyg.2021.689615

**Published:** 2021-08-26

**Authors:** Kyoko Yamamoto, Masanori Kimura, Miki Osaka

**Affiliations:** ^1^Department of Psychology, Kobe Gakuin University, Kobe, Japan; ^2^Department of Psychological and Behavioral Sciences, Kobe College, Nishinomiya, Japan

**Keywords:** sincere apology, instrumental apology, facial displays, gaze, self-monitoring

## Abstract

This study examines the effects of different types of apologies and individual differences in self-monitoring on non-verbal apology behaviors involving a server apologizing to a customer. Apologies divide into sincere apologies that reflect genuine recognition of fault, and instrumental apologies, made for achieving a personal goal such as avoiding punishment or rejection by others. Self-monitoring (public-performing and other-directedness) were also examined. Fifty-three female undergraduate students participated in the experiment. Participants were assigned randomly to either a sincere apology condition or an instrumental apology condition. They watched the film clip of the communication between a customer and server and then role-played how they would apologize if they were the server. Participants’ non-verbal behavior during the role-play was videotaped. The results showed an interaction between the apology condition and self-monitoring on non-verbal behaviors. When public-performing was low, gaze avoidance was more likely to occur with a sincere apology than an instrumental apology. There was no difference when the public-performing was high. Facial displays of apology were apparent in the instrumental apology compared to the sincere apology. This tendency became more conspicuous with increased public-performing. Our results indicated that the higher the public-performing, the more participants tried to convey the feeling of apology by combining a direct gaze and facial displays in an instrumental apology. On the other hand, results suggest that lower levels of public-performing elicited less immediacy in offering a sincere apology. Further studies are needed to determine whether these results apply to other conflict resolution situations.

## Introduction

We apologize when we make a mistake or cause trouble to others. Several studies have addressed the effects of apology. A meta-analysis of 175 studies showed that an apology was one of the most powerful predictors of interpersonal forgiveness, greater than any demographic, personality, or relationship characteristic (Fehr et al., 2010). However, an apology is not always effective in resolving conflicts. Whether or not conflict resolution occurs generally depends on the perception of the apology as trustworthy, genuine, and sincere by the injured party (Takaku et al., 2001).

Apologies divide into two types according to their authenticity and strategic nature (Ohbuchi et al., 2003, 2006; Nakagawa and Yamazaki, 2005). One is a sincere apology, made from the heart in recognition of fault, which requires guilt, recognition of remorse, and acceptance of responsibility (Tavuchis, 1991; Schmitt et al., 2004; Ohbuchi, 2010). The other is an instrumental apology made for a purpose such as avoiding punishment or rejection by peers. An instrumental apology is made to achieve a goal. It does not involve recognizing guilt or accepting responsibility. Moreover, an apology is one of the interpersonal emotion regulations that reduce the negative emotions of others. Ohbuchi et al. (1989) suggested that apologies reduce negative emotions and inhibit aggression in injured parties. In interpersonal emotion regulation, instrumental emotion regulation, where people regulate the feelings of others to achieve their personal goals, is documented (Netzer et al., 2015; Niven et al., 2018), and an instrumental apology can be considered a part of the same. Nakagawa and Yamazaki (2005) revealed that instrumental apologies do not resolve conflicts because violations repeat when there is no acceptance of responsibility or awareness of guilt. However, in some cases, an instrumental apology is required. Several types of instrumental apologies can be observed: one type is when the apologist is responsible but does not admit it and apologizes superficially. Another type is when the apologist is not responsible but apologies to appease the emotions of others. The former could be rephrased as an insincere apology but not the latter. The latter was the focus of this study. Recently, consumer complaints have become a major social issue in Japan (Ikeuchi, 2010). For example, in a service industry such as a shop, a restaurant, and a call center, an apology may be unavoidable in response to an unreasonable claim from a customer. In this case, since there is no fault on the part of the service employees, an instrumental apology that is unaccompanied by acceptance of responsibility or recognition of guilt calms the customer’s anger or maintains a good impression of the service employees. Abeler et al. (2010) found that apologies, even if strategic, effectively withdraw negative customer evaluations rather than offering monetary compensation. In the service scenarios, both sincere and instrumental apologies can occur. Therefore, we focus on the difference between sincere and instrumental apologies using the server–customer relationship.

Ohbuchi (2010) suggested that an apology often accompanies non-verbal behaviors such as facial expression and bodily movement, and the emotion of the offender can be conveyed to the injured party only when accompanied by non-verbal behavior suitable for the words of apology. Several studies have shown that non-verbal displays of sadness or remorse facilitated the effects of apology. ten Brinke and Adams (2015) indicated that a display of sadness as a signal of sincerity enhanced the effectiveness of an apology, as opposed to smiling. Similarly, Tamura (2009) showed that the expression of sadness reduced the anger of the injured party compared with a happy appearance, and anger increased when happiness showed, even if it was accompanied by words of apology. Physical displays of remorse such as kneeling or crying received positive appraisals of the transgressor and satisfaction with the apology (Hornsey et al., 2019). A common finding of these studies was that apologies accompanied by non-verbal displays of sadness or remorse reduced the negative feelings of the injured party and facilitated a positive evaluation of the offender.

Although most research focused on the perception of apologetic behavior, actual non-verbal behaviors during the apology act have not been studied sufficiently. Do non-verbal behaviors differ between sincere apologies and instrumental apologies? The two types of apology may have different non-verbal behaviors because a sincere apology is expressed consistently with the feelings experienced, whereas an instrumental apology is not. A sincere apology requires recognizing guilt and attendant remorse (Tavuchis, 1991; Schmitt et al., 2004; Ohbuchi, 2010). Research on the facial expressions of social emotions found no typical facial expression of guilt using static facial expression photographs (Keltner and Buswell, 1996). Guilt, uncaptured by static facial expressions, is expressed *via* complex patterns including facial expression, gaze, posture, and tone of voice (Ekman et al., 1991). Although previous studies have not clarified what facial expressions manifest when guilt occurs, research on the apology reported that transgressors looked guilty when conveying sadness through facial expressions (Darby and Schlenker, 1989; Tamura, 2009). Therefore, the facial display of sadness is a component of a sincere apology. By contrast, an instrumental apology is considered a deceptive apology. Previous studies have found a longer duration in false expressions than genuine expressions of emotions (e.g., Hess and Kleck, 1990; Frank et al., 1993). ten Brinke et al. (2012) comparing genuine remorse with fabricated remorse showed that deceptive descriptions of remorseful emotions are associated with the facial expression of the frontal region (i.e., the forehead area) in attempts to express falsified sadness. It appears that an apology accompanies a facial display of sadness more frequently in an instrumental apology than in a sincere apology.

Research on deception has often dealt with the gaze. Gaze behavior might differ between a sincere apology and an instrumental apology. In general, gaze aversion is considered a reliable signifier of deceit (Global Deception Research Team, 2006). Previous research suggests that gaze aversion increases the perception of deception on the part of the injured party and, therefore, lowers the perception of credibility, regardless of whether the speaker is lying or not (Vrij and Semin, 1996; Strömwall and Granhag, 2003; Reinhard and Sporer, 2008; Bogaard et al., 2016). The opposite is true; liars may make more eye contact than truth-tellers in a conscious attempt to appear convincing (Vrij, 2008). Therefore, people making an instrumental apology would affect more gaze behaviors to convince and counter a falsified apology. Besides, Yu et al. (2017) showed that participants fixated less on the partner’s eyes in high guilt conditions than low guilt conditions. Considering that the feeling of guilt aroused by sincere apologies is associated with gaze aversion, it infers that instrumental apologies are more frequently gaze-focused than sincere apologies.

Personality may also be involved in making an appropriate apology according to the situation. Schlenker and Weigold (1992) suggest that interest in self-presentation and impression management is part of a motive for apologizing. In this study, we focus on self-monitoring, defined as a personality trait and an ability to regulate expressive behaviors to accommodate social situations (Snyder, 1974). Individuals with high self-monitoring are more concerned about the situational appropriateness of social behavior and are more likely to adapt their behavior according to the situation. Friedman and Miller-Herringer (1991) showed that people with a high level of self-monitoring are better at hiding pleasure than people with low levels in scenarios where a pleased expression is inappropriate. Self-monitoring was positively associated with the level of surface acting in emotional labor that expresses emotions that differ from the subjective emotional states (Buckner and Mahoney, 2012; Scott et al., 2012). Therefore, differences in self-monitoring have a significant impact in the instrumental apology where subjective feelings and expressed emotions differ.

This study investigates the differences in non-verbal behaviors associated with a sincere apology and an instrumental apology through the role-play of apologizing to customers as a server. Self-monitoring effects were also examined. We measured gaze behavior and facial displays of sadness, which relate to an apology. Furthermore, we measured subjective indices to study the differences in acceptance of responsibility and the subjective feelings between the two types of apologies. Based on the above discussion, this study explores the following three hypotheses:

*Hypothesis 1*: Gaze aversion is more likely to occur in a sincere apology than in an instrumental apology.
*Hypothesis 2*: Instrumental apology facilitates facial displays of sadness more than a sincere apology.
*Hypothesis 3*: Compared with those in sincere apology, the facilitation of apologetic facial displays in instrumental apology is greater in the high self-monitor than in the low self-monitor.

## Materials and Methods

### Participants

Fifty-three Japanese female students (mean age = 19.85 ± 1.03 years old) participated in this study and were assigned randomly to a sincere apology condition (27 participants) or an instrumental apology condition (26 participants). The author’s institution ethically reviewed this study.

### Experimental Stimuli

The apology scenario took place in a cafe, where we filmed the interaction between a server and a customer. The participants were instructed to watch the video as if they were the server. Female students played the role of the server and the customer to exclude sex difference effects and its interaction. The film clip showed that when a server offered a glass of water to the customer, the water spilled over and got on the customer’s clothes, making the customer angry. The stimuli ended with a scene of an angry customer. Participants had to role-play an apology as a continuation of that scene. This film clip was recorded from a back angle, except when the customer got angry and her face displayed in close-up. In the condition of a sincere apology, it was the server’s fault. In the condition of an instrumental apology, it was the customer’s fault whose hand slipped and caused the spill.

### Questionnaire

#### Manipulation Check

For manipulation checks, we asked participants about the degree to which they thought the fault was the responsibility of the “server” and the “customer” based on a 7-point Likert scale (1 = not at all, 7 = very much).

#### Subjective Emotion

We asked participants to indicate their feelings from the server’s perspective in the film clip. We wanted to confirm whether the intended emotion was aroused by each apology condition after the stimulus viewing. Participants rated 16 items using a 5-point Likert scale: “guilt,” “remorse,” “anger,” “loneliness,” “happiness,” “impatience,” “upset,” “disgust,” “surprise,” “sadness,” “antipathy,” “embarrassment,” “the feeling of being injured by the customer,” “responsibility,” “forgiveness from the customer,” and “forgiveness for the customer” (1 = not at all, 5 = extremely).

#### Self-Monitoring Scale

Self-monitoring was measured using a scale by Iwabuchi et al. (1996), the Japanese version of Snyder’s (1974) self-monitoring scales. It consists of 25 items (using a 5-point Likert scale, with 1 = strongly disagree and 5 = strongly agree). We conducted a factor analysis on the self-monitoring items because the factor structure of the self-monitoring scale varies among previous studies (Lennox, 1988). Early factor analytic research yielded three factors: acting, extraversion, and other-directedness (Briggs et al., 1980); however, later studies found two dimensions (Briggs and Cheek, 1988). We extracted two factors: public-performing (α = 0.75) and other-directedness (α = 0.74). This two-factor approach is dominant in reviews of the self-monitoring construct demonstrating the predictive utility (Gangestad and Snyder, 2000). Public-performing captures aspects of acting and extraversion, indicating an ability to change behavior to function well in social situations. Other-directedness reflects attention to other people’s expectations and motivates willingness to mask true feelings in pleasing others.

### Procedure

On arrival at the laboratory, participants were told that they were participating in a study examining the psychology of the server in customer service. They were also instructed that they would be videotaped while role-playing a server in a cafe. Those who agreed to participate were required to sign a consent form. Next, participants were asked to respond to the self-monitoring scale. Participants were told they would watch the film clip on the communication between the customer and the server who would enact the role after watching. After watching the film clip, participants completed the manipulation check and the subjective emotion questionnaire. Then, the experimenter asked participants to play the role of server and apologize to the customer as a continuation of the film clip. Participants were asked to wear the apron that the server wore in the film clip to get into the role. The verbal expression of the apology was unified into “I’m terribly sorry. I’ll bring a hand towel right away.” After 1 min of practice, participants apologized as the server and recorded by a video camera. Finally, participants were debriefed and viewed a pleasant video clip to reduce any negative feelings that might have emerged from the experiment.

### Coding of Expressive Behaviors

Two coders recorded the occurrences of gaze and turning one’s face toward the customer (video camera) using the event recorder “Sigsaji” (Arakawa and Suzuki, 2004), created using Microsoft Excel add-in functionality. It had a time resolution of 0.5 s. Turning one’s face is coded based on the server facing the front of the video camera where the customer was expected to be, and gaze based on the eyes. The durations of turning one’s face and gaze were then computed. The inter-coder reliability of these indices was α = 0.86–0.87. Mean coder scores were used in the analyses of these indices. The action units (AUs) of facial displays were coded using Open Face (Baltrušaitis et al., 2015, 2018), which is an automatic facial behavior analysis toolkit. The duration of AU1 (inner brow raiser), AU2 (outer brow raiser), AU4 (brow lowerer), and AU15 (lip corner depressor), which consists of sadness facial displays, were used for the analyses.

## Results

### Manipulation Check

All participants said the instructed apology sentence in the role-play. To assess the effectiveness of manipulating the apology condition, we performed a *t*-test on the manipulation check items to assess the degree of responsibility of the server and customer. The results showed that the responsibility on the part of the server was rated higher in the sincere apology condition [sincere apology *M* = 5.07, SD = 1.44; instrumental apology *M* = 1.65, SD = 0.98, *t*(51) = 10.08, *p* < 0.001, *d* = 2.82], whereas the responsibility on the part of the customer was rated higher in the instrumental apology condition [sincere apology *M* = 1.81, SD = 0.88; instrumental apology *M* = 5.69, SD = 1.01, *t*(51) = 14.92, *p* < 0.001, *d* = 4.18]. In the following analyses, to clarify the difference between conditions, we extracted participants who had five or more points of the degree of the server/customer being the responsible party in the condition of sincere apology/instrumental apology. Twenty participants in the sincere apology condition and 24 participants in the instrumental apology condition remained.

### Subjective Emotion

To examine emotional differences between the apology conditions, we conducted a *t*-test on each item of subjective feelings (Table 1). Feelings of guilt, remorse, responsibility, sadness, and embarrassment, scored higher in the sincere apology condition. Those of anger, antipathy, and feeling injured by the customer scored higher in the instrumental apology condition. These results also confirmed that the apology manipulation was effective.

**TABLE 1:** Mean scores of subjective emotions.

	Sincere		Instrumental			
	*M*	SD	*M*	SD	*t* (42)	*d*
Guilt	4.50	0.61	2.38	0.88	9.17***	2.83
Remorse	4.60	0.50	2.67	1.09	7.30***	2.26
Responsibility	4.65	0.49	2.96	1.16	6.08***	1.18
Sadness	3.50	1.19	2.13	1.33	3.58**	1.11
Embarrassment	3.35	1.35	2.42	1.18	2.45*	0.76
Anger	2.00	0.97	3.38	0.88	4.93***	1.53
Antipathy	2.35	1.31	3.46	1.06	3.10**	0.96
The feeling of being injured by the customer	3.30	1.03	4.13	0.90	2.83**	0.88
Loneliness	2.30	1.30	1.63	0.92	2.01	0.62
Happiness	1.00	0.00	1.13	0.34	1.65	0.53
Impatience	4.70	0.47	4.46	0.51	1.62	0.50
Upset	4.45	0.69	4.54	0.51	0.51	0.15
Disgust	3.60	1.14	3.54	1.10	0.17	0.05
Surprise	3.50	1.24	3.83	1.09	0.95	0.29
Forgiveness for the customer	2.90	0.91	2.71	1.20	0.59	0.18
Forgiveness from customer	2.20	0.89	2.29	1.16	0.29	0.09

*p < 0.05, **p < 0.01, ***p < 0.001.

### Expressive Behaviors

Hierarchical multiple regression analysis examined whether the apology condition (0 = sincere apology condition, 1 = instrumental apology condition) and self-monitoring (public-performing/other-directedness) predicted each non-verbal behavior. We deployed the apology condition and one of the self-monitoring subscales in the first step, followed by the interaction of the apology condition and one of the self-monitoring subscales. HAD (Shimizu, 2016), created using Microsoft Excel add-in functionality, was then used for hierarchical multiple regression analysis.

#### Gaze and Turning One’s Face

**FIGURE 1:**
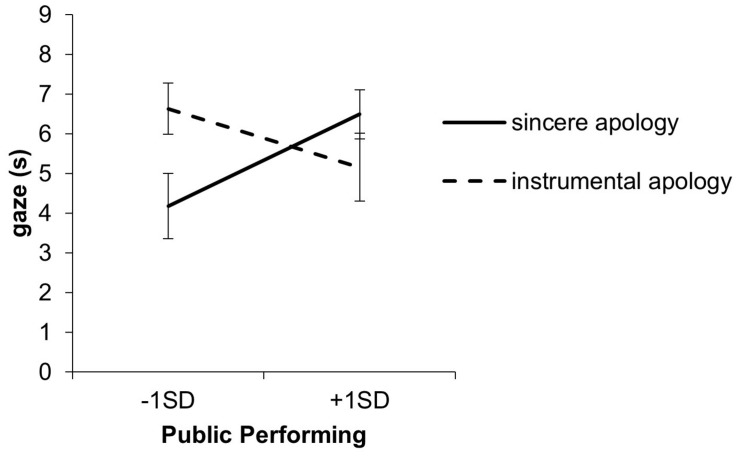
Interaction of the apology condition and public-performing for the duration of gaze. Error bars represent standard errors.

In the hierarchical regression analysis for the duration of gaze, the effects of condition and public-performing were not significant in Step 1. The apology condition × public-performing interaction was significant in Step 2 (*R*^2^ = 0.16, *b* = −3.06, SE = 1.26, β = −0.37, *p* = 0.02; Figure 1). A simple slope analysis revealed that public−performing significantly predicted gaze in the sincere apology condition (*b* = 1.87, SE = 0.83, β = 0.46, *p* = 0.03), but not in the instrumental apology condition (*b* = −1.19, SE = 0.96, β = −0.30, *p* = 0.22). Also, the instrumental apology condition showed longer duration of gaze than the sincere apology condition for those low in public-performing (*b* = 2.46, SE = 1.04, β = 0.54, *p* = 0.02), but not in those high in public-performing (*b* = −1.33, SE = 1.06, β = −0.29, *p* = 0.21).

**FIGURE 2:**
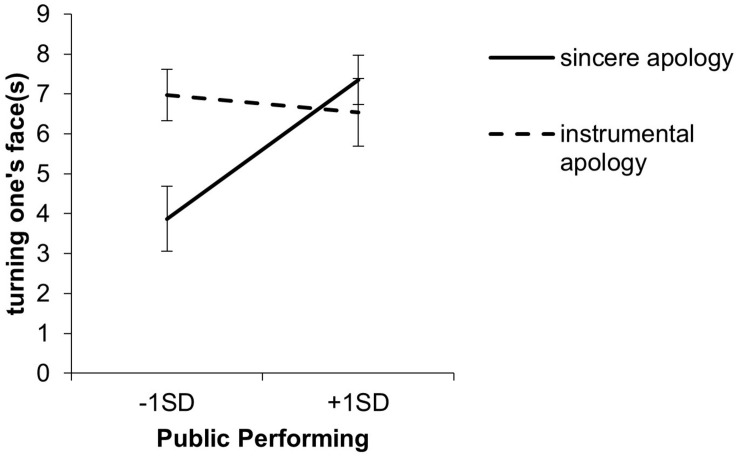
Interaction of the apology condition and public-performing for the duration of turning one’s face. Error bars represent standard errors.

For the duration of turning one’s face, the effect of public-performing was significant in Step 1 (*R*^2^ = 0.13, *b* = 1.46, SE = 0.66, β = 0.34, *p* = 0.03). In Step 2, the apology condition × public-performing interaction (*R*^2^ = 0.26, *b* = −3.16, SE = 1.25, β = −0.36, *p* = 0.02; Figure 2) was significant. A simple slope analysis revealed the same pattern of gaze (the sincere apology condition: *b* = 2.81, SE = 0.82, β = 0.66, *p* = 0.001, the instrumental apology condition: *b* = −0.35, SE = 0.95, β = −0.08, *p* = 0.72, low public-performing: *b* = 3.10, SE = 1.03, β = 0.68, *p* = 0.005, high public-performing: *b* = −0.81, SE = 1.05, β = −0.17, *p* = 0.45).

In the analysis of the apology condition × other-directedness, there were no significant effects (Table 2).

**TABLE 2:** Hierarchical multiple regression analysis predicting gaze and turning one’s face from apology condition and other-directedness.

	Gaze		Turning one’s face	
Predictor	Δ *R*^2^	β	Δ *R*^2^	β
Step 1	0.02		0.03	
Condition		0.11		0.17
Other-directedness		–0.10		–0.09
Step 2	0.004		0.003	
Condition		0.12		0.17
Other-directedness		–0.09		–0.08
Condition×		–0.07		–0.06
Other-directedness				
Total *R*^2^	0.02		0.03	

*N = 42. Condition: 0 = sincere apology, 1 = instrumental apology.*

#### AUs

Table 3 shows the results of hierarchical regression analysis predicting each of the AUs from the apology condition and public-performing. For the duration of AU4, the effect of the apology condition was significant in Step 1 (*R*^2^ = 0.14, *b* = 1.53, SE = 0.70, β = 0.34, *p* = 0.04). The apology condition × public-performing interaction was not significant in Step 2. A similar pattern, although it did not reach a significant level, was observed in AU1 and AU2. For the duration of AU15, there were no main effects in Step 1. In Step 2, the apology condition × public-performing interaction was significant (*R*^2^ = 0.15, *b* = 0.70, SE = 0.34, β = 0.32, *p* = 0.04). Simple slope analysis revealed that public-performing significantly predicted the duration of AU15 in the instrumental apology condition (*b* = 0.61, SE = 0.26, β = 0.57, *p* = 0.02), but not in the sincere apology condition (*b* = −0.09, SE = 0.22, β = −0.09, *p* = 0.68). Also, the instrumental apology condition showed a longer duration than the sincere apology condition for those high in public-performing (*b* = 0.68, SE = 0.28, β = 0.56, *p* = 0.02), but not in those low in public-performing (*b* = −0.19, SE = 0.28, β = −0.15, *p* = 0.51).

**TABLE 3:** Hierarchical multiple regression predicting each of AUs from the apology condition and public-performing.

	AU1		AU2		AU4		AU15	
Predictor	Δ *R*^2^	β	Δ *R*^2^	β	Δ *R*^2^	β	Δ *R*^2^	β
Step 1	0.09		0.15^†^		0.14^†^		0.06	
Condition		0.31^†^		0.29^†^		0.34*		0.20
Public performing		0.10		−0.18		−0.08		0.19
Step 2	0.10		0.01		0.05		0.10*	
Condition		0.31^†^		0.29^†^		0.34*		0.21
Public performing		0.12		−0.17		−0.04		0.24
Condition×		0.09		0.08		0.22		0.32*
Public performing								
Total *R*^2^	0.10		0.15^†^		0.18^†^		0.15^†^	

N = 42. Condition: 0 = sincere apology, 1 = instrumental apology. ^†^p < 0.10; *p < 0.05.

Table 4 shows the results of hierarchical regression analysis predicting each of AUs from apology condition and other-directedness. In the analysis of the apology condition × other-directedness, the main effect of the apology condition was significant in AU2 (*R*^2^ = 0.18, *b* = 0.66, SE = 0.25, β = 0.38, *p* = 0.02) and AU4 (*R*^2^ = 0.15, *b* = 1.64, SE = 0.69, β = 0.36, *p* = 0.03) in Step 1. The apology condition × other-directedness was not significant for the duration of any AU.

**TABLE 4:** Hierarchical multiple regression analysis predicting each of AUs from the apology condition and other-directedness.

	AU1		AU2		AU4		AU15	
Predictor	Δ *R*^2^	β	Δ *R*^2^	β	Δ *R*^2^	β	Δ *R*^2^	β
Step 1	0.09		0.18*		0.13^†^		0.03	
Condition		0.30^†^		0.38*		0.36*		0.13
Other-directedness		−0.11		−0.26^†^		−0.02		0.08
Step 2	0.002		0.000		0.02		0.03	
Condition		0.30^†^		0.39*		0.35*		0.12
Other-directedness		−0.10		−0.26^†^		−0.05		0.16
Condition×		−0.04		−0.02		0.15		0.17
Other-directedness								
Total *R*^2^	0.09		0.18^†^		0.15^†^		0.06	

N = 42. Condition: 0 = sincere apology, 1 = instrumental apology. ^†^p < 0.10; *p < 0.05.

## Discussion

This study examined the effects of the type of apology (a sincere/an instrumental apology) and individual differences of self-monitoring on the non-verbal behaviors of apology focusing on the server–customer relationship. We also examined whether individual differences in self-monitoring affect these non-verbal behaviors.

The present study tested three hypotheses. Hypothesis 1 was that gaze aversion was more likely to occur in a sincere apology than in an instrumental apology. Regarding gaze and turning one’s face, the main effect of the apology condition was unseen. However, the interaction between apology conditions and public-performing of self-monitoring was observed such that the instrumental apology conditions showed a longer duration of gaze and turning the face than the sincere apology conditions when public-performing was low. Therefore, Hypothesis 1 was supported only when the public-performing self-monitoring was low.

When the public-performing self-monitoring was high, there was no difference in turning one’s face and gaze between the sincere apology and the instrumental apology. In other words, a high-level public-performing person can respond similarly to two types of situations. Consistent with ideas expressed by other researchers (Snyder, 1974; Friedman and Miller-Herringer, 1991; Buckner and Mahoney, 2012; Scott et al., 2012), our findings suggest that high self-monitors are better at controlling their behavior according to the situation. This result is also consistent with research suggesting that people believe that an averted gaze gives an impression of deceptiveness (Vrij and Semin, 1996; Strömwall and Granhag, 2003; Reinhard and Sporer, 2008; Bogaard et al., 2016). In addition, Yu et al. (2017) suggested that eye contact with the injured party in guilt situations may heighten the emotional arousal of the transgressor. It seemed that people with high public-performing could regulate such unpleasant arousal and look at the customer. Our results suggest that a high self-monitoring public-performing person tried to convey the heartfelt apology by looking at the other person in both types of apology.

Another explanation for this result is that the person with high public-performing self-monitoring intended to see the customer reaction to the apology regardless of apology conditions. The eyes are the windows to the mind and help to infer the mental states of others (Kendon, 1967; Calder et al., 2002; Khalid et al., 2016). In this study, however, the role-play apology was made to the video camera, and there was no real customer involved. It suggests that the expression directed toward others appears even by imagining the existence of others (Fridlund, 1991, 1994; Jakobs et al., 1999, 2001), in this study. Future research is needed to examine this issue using the situation in which the person materially exists in front of the apologizer.

Hypothesis 2 was that the instrumental apology facilitated more extended facial displays of an apology than a sincere apology. In support of this hypothesis, the facial display duration in the upper half of the face (AU1, AU2, and AU4) was longer in the instrumental apology than in the sincere apology. These findings are consistent with the study of ten Brinke et al. (2012), which suggested that the frontal muscle activities were facilitated more with fabricated remorse compared with sincere remorse. It also corresponds with the finding that intentional facial expressions last longer than spontaneous facial expressions (Ekman and Friesen, 1982; Ekman, 1988; Hess and Kleck, 1990; Hill and Craig, 2002; Ekman and O’Sullivan, 2006). An alternative explanation for the results is that AU1, AU2, and AU4 reflect the expression of anger. AU1, AU2, and AU4 are not only expressions of guilt or sadness but expressions related to anger and surprise. As the conditions for instrumental apologies aroused anger and antipathy, it cannot be ruled out that the increased activity of the AU1, AU2, and AU4 was an expression corresponding to these emotions.

Hypothesis 3 was that compared with those in sincere apology, the facilitation of apologetic facial displays in instrumental apology is greater in the high self-monitor than in the low self-monitor. In AU15 (lower the corners of the mouth), an interaction between the apology conditions and public-performing self-monitoring was observed, such that the instrumental apology conditions were found to lower the corners of the mouth compared to sincere apology conditions when public-performing was high. On the other hand, as mentioned above, only the main effect of the apology condition was significant in AU1, AU2, and AU4. Therefore, Hypothesis 3 was supported only in AU15. AU1, AU2, and AU4 belong to the upper half of the face, and AU15 belongs to the lower half face. It is pointed out that the motor controls of the upper and lower halves of the face are independent (Ekman and Friesen, 1969; Ekman et al., 1980; Ekman, 1985, 1989; Levenson et al., 1990). The facial displays around the mouth are easier to manipulate intentionally than in the upper half (Ross et al., 2016). It infers that the person with high public-performing facilitated the expression around the mouth by intentionally conveying the feeling of apology. In addition, AU1, AU2, and AU4 are also moving parts in feeling anger. Therefore, there is a risk that using these expressions in the instrumental apology will be regarded as anger from the customer’s point of view. Smith (2008) pointed out that sadness is a component of remorse, but anger is generally considered to be discordant with feelings of regret. It suggests that people with high public-performing expressed apology by clearly expressing AU15 and tried to prevent inappropriate anger feelings from being misunderstood in the instrumental apology.

To summarize the above results of non-verbal behaviors, high public-performing in self-monitoring performed gaze behavior is equivalent to that in the condition of instrumental apology under sincere apology and facilitated the expression of AU15 in the instrumental apology. It suggests that direct gaze enhances recognition of emotional expressions compared to an averted gaze (Vuilleumier and Pourtois, 2007; Bindemann et al., 2008). These results indicate that people with high public-performing tried to convey an apology to a customer by combining direct gaze and remorseful facial display in a given situation even though they were not at fault.

Among the subscales of self-monitoring, public-performing influenced non-verbal behavior of apology, but other-directedness had little effect. While public performing (extraversion and acting) is consistent with the concept of self-monitoring defined as a personality trait and an ability to regulate expressive behaviors to accommodate social situations, as proposed by Snyder (1974), the other-directedness is orthogonal to the public-performing dimension and may not apply to the definition of self-monitoring (Wilmot, 2015). In line with this, Riggio and Friedman (1982) showed that the extraversion of self-monitoring correlates positively with non-verbal expressiveness but not with other-directedness. Wilmot (2015) also noted that acting and extraversion subscales included in public-performing served as the output indicators, and the other-directedness subscale served as the input indicator. These differences in the properties of public-performing and other-directedness may have influenced the results.

The result of self-monitoring and deception seems to be generally widespread in extraversion. People with a high level of extraversion are more likely than introverts to practice deceit, and this tendency is apparent even when controlling for extroverts’ level of interaction with others (Kashy and DePaulo, 1996; Weiss and Feldman, 2006). Also, according to the type of lies, people with high self-monitoring were more likely to tell selfish lies (Mcleod and Genereux, 2008). The instrumental apology can be regarded as a selfish lie to protect oneself or one’s organization, consistent with the public-performing results of this study.

The current study indicates that the non-verbal behaviors of the apology differed depending on the type of apology and individual differences in public performing of self-monitoring. We use the situation in which the server was forced to apologize despite not being at fault as a scenario of the instrumental apology. It is necessary to examine whether the results of this study apply to other apology situations. There may be cases in which an offender apologizes superficially without admitting their responsibility. In this case, more selfish motives are expected than the situation used in this study. Also, the verbal apologies were unified in this study to focus on non-verbal behaviors. However, there is a possibility that the verbal behavior used for apology differs depending on the types of apology and individual differences in self-monitoring. Since the effects of apologies may differ depending on the combination of verbal and non-verbal behavior, it is also desirable to consider verbal behavior. It is also questionable whether the service and personal relationship apologies are the same. Acquiring a good reputation is common in both personal and service situations. Although personal relationships tend to continue into the long term, service relationships tend to end with only temporary exchanges. Therefore, it is necessary to examine an apology in the personal relationship in which the motivation for maintaining the relationship is high. Further research is needed to replicate the current findings by using different apology situations.

Some limitations exist in this study. First, the effect size was small for the apology condition. The impact of the experimental manipulation of role-playing the apology may have been weak. Guilt and remorse were rated higher for the sincere apology. Anger and upset were rated higher for the instrumental apology in the subjective emotions, but these ratings may be influenced by demand characteristics rather than a subjective emotional experience. Second, the non-verbal behavior obtained in role-playing may be different from spontaneous expression. We must examine this issue by using an experimental situation wherein spontaneous expressions are more easily detected, such as some rigged game scenarios that require winning participants to apologize to their losing opponents. Third, this study focused only on communication between females. Sex differences in facial expressions have frequently been reported in the literature (e.g., Hall, 1984). It is not clear whether this result can be extended to males or male–female combinations. Further study is needed on these above points. Fourth, the cultural factors might affect the results of this study conducted in Japan. It has been suggested that the Japanese have an interdependent view of self and are more likely to apologize during interpersonal conflicts (Ohbuchi et al., 2003). Therefore, examining whether the results of this study also apply to other cultures is necessary.

There are other important research questions concerning two types of apology. First, whether differences in non-verbal behaviors by the types of apology and self-monitoring impact the effectiveness of apologies. We examined the non-verbal behaviors expressed during an apology, which have not been focused on in previous studies; however, the perception of these non-verbal behaviors is also an important issue to be examined. Research on the effectiveness of non-verbal behaviors during an apology has been examined by comparing expressions of sadness or remorse with the lack of these expressions or smiles (Tamura, 2009; ten Brinke and Adams, 2015; Hornsey et al., 2019). Hornsey et al. (2019) suggested that an apology accompanied by a non-verbal expression of remorse increases positive appraisals but has little effect on forgiveness. It is necessary to examine the influence of facilitation or inhibition of the non-verbal behaviors on the effectiveness of the two types of apology in the future.

Second, how does the burden of an instrumental apology affect the apologizer? Research on deception indicated that it is harder to express emotions differing from internal emotional states than simply hiding them. This tendency is stronger in the case of negative emotions (Porter and ten Brinke, 2008). It is true of instrumental apologies. However, Scott et al. (2012) suggest that the effects of surface acting on a decrease in job satisfaction and work withdrawal were weaker when self-monitoring was high. Therefore, high self-monitoring may be able to cope with the burden of instrumental apologies. Further examination would shed light on the burden of forced instrumental apology in such situations.

## Data Availability Statement

The raw data supporting the conclusions of this article will be made available by the authors, without undue reservation.

## Ethics Statement

The studies involving human participants were reviewed and approved by Institutional Review Board, Kobe College. The patients/participants provided their written informed consent to participate in this study.

## Author Contributions

All authors contributed to conception of the study. KY performed facial expression analysis and statistical analysis, and drafted the manuscript. MK participated in the design of the study and helped to draft the manuscript. MO conceived of the study, and performed the experiment and coding of expressive behaviors. All authors read and approved the submitted version.

## Conflict of Interest

The authors declare that the research was conducted in the absence of any commercial or financial relationships that could be construed as a potential conflict of interest.

## Publisher’s Note

All claims expressed in this article are solely those of the authors and do not necessarily represent those of their affiliated organizations, or those of the publisher, the editors and the reviewers. Any product that may be evaluated in this article, or claim that may be made by its manufacturer, is not guaranteed or endorsed by the publisher.
